# A Severe Case of Biparietal Thinning in a Medieval Skull From a Northern Italy Necropolis

**DOI:** 10.1097/SCS.0000000000007929

**Published:** 2021-07-13

**Authors:** Enrica Tonina, Omar Larentis, Chiara Tesi, Roberta Fusco, Monica Campagnolo, Marta Licata

**Affiliations:** ∗ Department of Biotechnologies and Life Sciences, Centre of Research in Osteoarchaeology and Paleopathology; † Department of Biotechnologies and Life Sciences, Insubria University, Italy.

**Keywords:** Biparietal thinning, Medieval Age, north Italy, osteoporosis, paleopathology

## Abstract

This study aims at presenting a case of symmetrical and bilateral thinning observed in a skull belonging to the skeleton of a mature woman from the medieval cemetery of Caravate (north Italy). Macroscopical, radiological, and histological analyses were performed to investigate the condition. The analyses allowed us to detect a progressive loss of both the outer table and the diploe, and the sparing of the inner table. As a controversial condition in the clinical and paleopathological literature, this case poses some difficulties in discussing the differential diagnosis. However, the sex determination, estimation of the age-at-death and different characteristics observed at the level of the postcranial bones, in particular the fractures recorded on different vertebral bodies, allowed us to correlate the biparietal thinning found in this subject to ageing and osteoporosis.

Symmetrical and bilateral thinning (SBT) of the parietal bones is a controversial condition discussed both in palaeopathological and clinical literature. Indeed, in the past, SBT was described as a nonmetric trait, an anatomical variation, and a developmental anomaly. Although its etiology is still unknown, some authors have considered biparietal thinning as a pathological lesion.^1,2^

In modern clinic, it is particularly recorded in individuals aged over 60 years and registers an estimated incidence between 0.4% and 2.37%.^3^ Its incidence is probably underestimated, since the detection of this condition, mostly asymptomatic, usually occurs randomly during diagnostic analyses. However, this condition shows no connection with phenotype or predisposition for a specific geographic area.^4^

From a macroscopic and a radiological point of view, it is recognized as a flat oval or quadrilateral alteration.^5^ Microscopically, SBT is characterized by the erosion, loss of substance, and remodeling of the external table and diploe, and an intact inner table.^1,2,4,6,7^

Today, it is still not possible to clarify the origin of this type of lesion. Even if authors have elaborated several hypotheses linked to its etiology as senescence, trauma, muscular traction, inflammatory reaction, none of these can explain its symmetry, its localization, or its age/sex predilection.^1–8^

Palaeopathological literature shows several findings of SBT. The most ancient case has been documented in India, in a skull from the Bronze Age.^9^ The recorded cases come from different areas of the world. However, researches are especially concentrated on the Egyptian sample. In particular, one of the most extensive analysis on SBT find a prevalence of the condition in 4.9% of cases in a sample of about 1000 subjects^10,11^; in Breitinger study,^12^ 4 skull out of 27 specimens (14.4% of prevalence) showed SBT; in Tomb 32 of the necropolis of Thebes, the occurrence of SBT was up to 30% (in a sample of 312 adults).^13,14^

The development of bioarchaeological sciences, through the excavations of other necropolis and the study of new osteoarchaeological samples, will certainly bring to light other evidence of this condition also in different places of the world. Moreover, the advanced observational methods applied on ancient human remains will allow discovering pathological signs from the past until now unknown and enriching the diagnostic analysis in the clinical experience.

In this paper, we present a case of SBT recorded on the skeleton of a woman discovered in a medieval Italian cemetery ^15,16^ and discuss this condition from the morphological, radiological, and histological point of view.

## MATERIALS AND METHODS

From 2001 to 2019, archaeological investigations conducted in the medieval site of the church of Saint Agostino in Caravate (Varese, north Italy) allowed us to discover a funeral area dated back to High Middle Ages, exploited approximately from the 11th century.^17^

**FIGURE 1:**
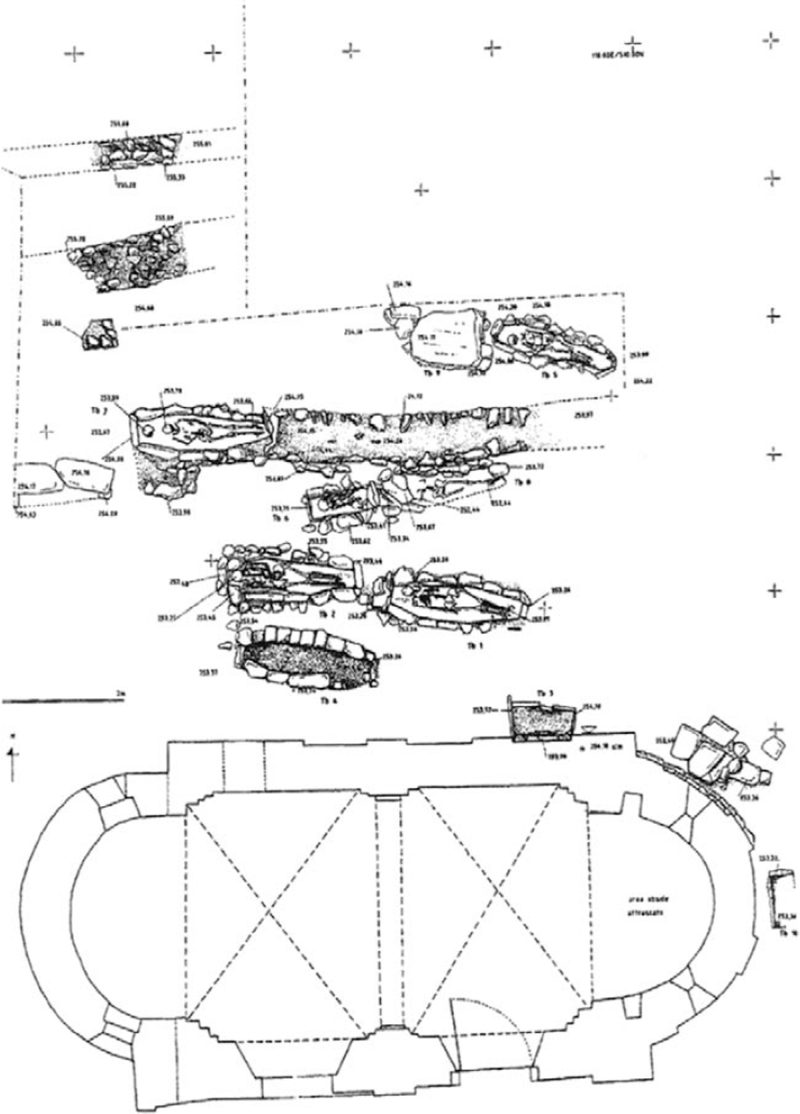
Planimetry of Caravate necropolis.

Several archaeological phases have been recovered and, until now, 20 structured tombs, 2 of them reused as common ossuary, have been brought to light (Fig. 1).^18,19^

The bioarchaeological study conducted by the researchers of the Centre of Research of Osteoarchaeology of the cemetery of Saint Agostine allowed us to discover different anthropological aspects of a medieval population of North-Western Lombardy.^20,21^

**FIGURE 2:**
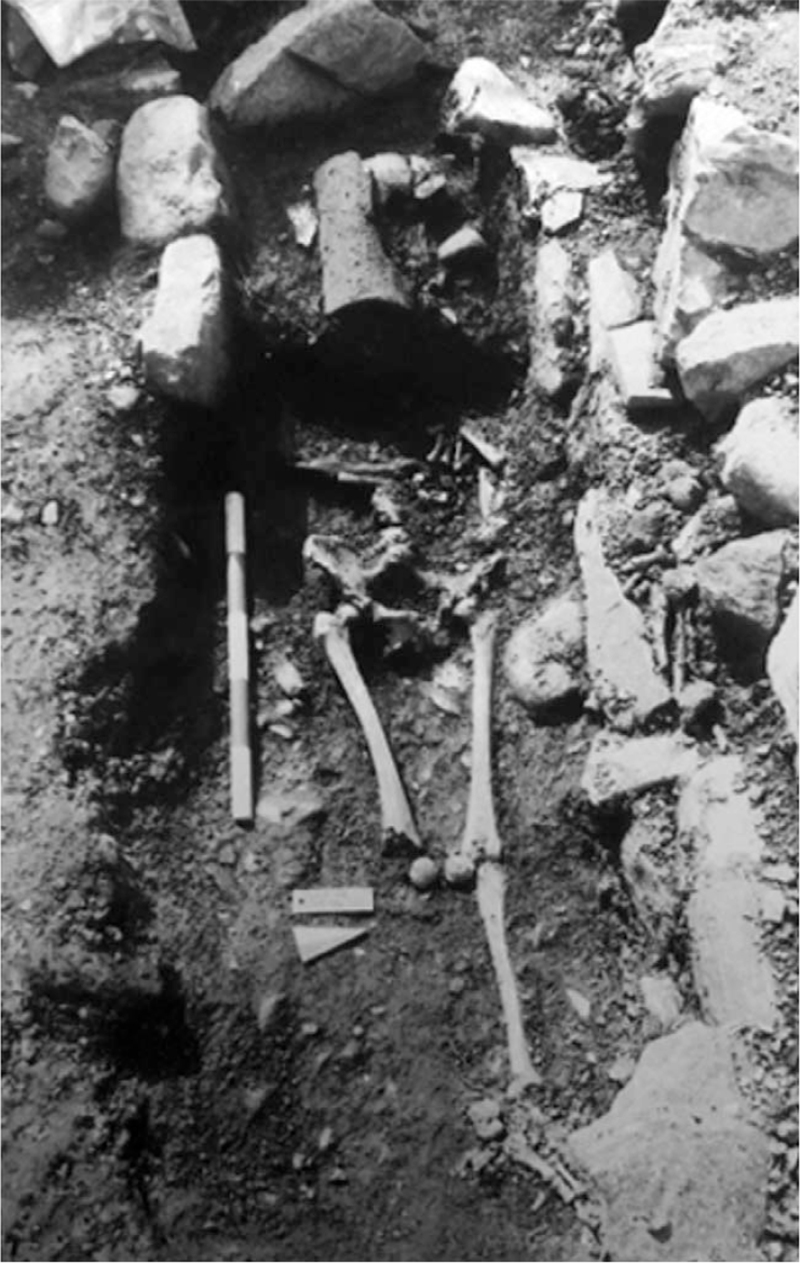
Tomb 8 of the cemetery area of Sant’Agostino in Caravate.

The skeleton under investigation laid inside an anthropomorphic tomb-oriented East-West (tomb 8). The upper portion from the skull to the pelvis lay on a raised level. The upper limbs were crossed on the abdomen whereas the lower ones were stretched parallel to each other. The column maintained its anatomical connection and the pubic symphysis was not disjoint. The kneecaps were fallen medially. The taphonomic characteristics of the inhumation, in association with the position of the tile covering the cranium, suggest a deposition in full space (Fig. 2).

**FIGURE 3:**
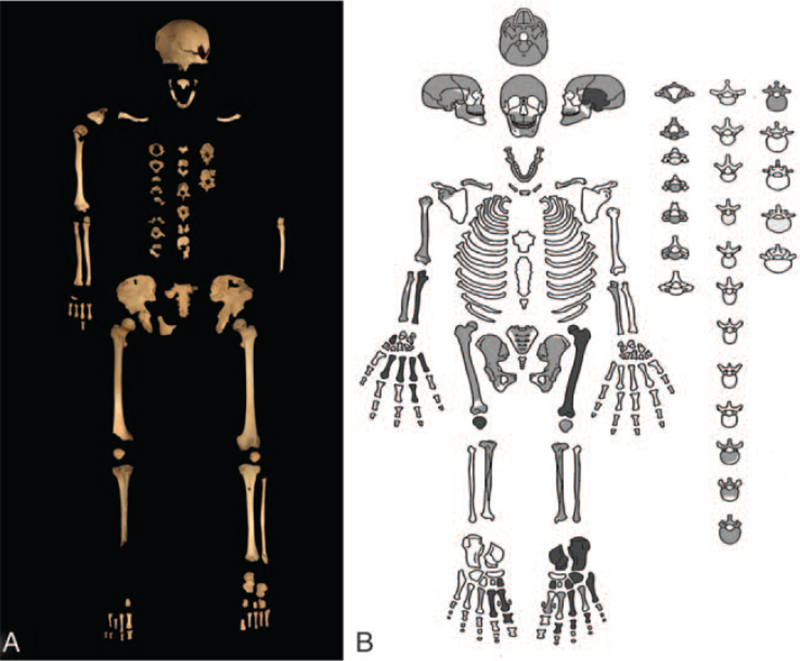
(A) state of preservation of the skeleton and (B) diagrammatic illustration of its state of its representation.

In order to reconstruct the biological profile of the skeleton (Fig. 3A-B) we proceeded with the anthropological analysis. Sex determination was carried out by using macroscopic methods, through the observations of morphological features of the skull^22^ and the morphology^23,24^ and measures of the pelvis.^25^ Age at death was estimated through the analysis of cranial sutures,^26^ degenerative changes of the pubic symphysis and senescence of the auricular surface.^27,28^ Bone measurements and anthropometric index were calculated following Martin and Saller.^29^ We applied Trotter and Gleser formulae to obtain the determination of stature.^30^ To reveal pathological conditions, a depth observation of all bone segments was conducted.

Radiographic analyses were carried out at the Gaetano and Piera Borghi Foundation (Brebbia, VA). For the realization of the radiographic images, a DR Fujifilm machine was used with an exposure (100 ms) at 55 kV to 100 mA. Computer tomography analyses were performed with a Hitachi Eclos 16 machine with 90 to 120 kV, 100 to 400 mA exposure. Histological investigations were also carried out. The bone tissue sample was incorporated in Technovit 7200 resin. The thin section of 300 to 400 μm was observed under an Aristoplan Leitz optical microscope under normal light.

## RESULTS

The skeleton of tomb 8 belonged to a woman with an estimated age at death of 45 to 55 years and 152 to 155 cm tall.

The anthropometric measurements of the skull highlighted a brachycranic shape, and the postcranial indexes showed a general weakness of the biomechanical structures of the limbs.

**FIGURE 4:**
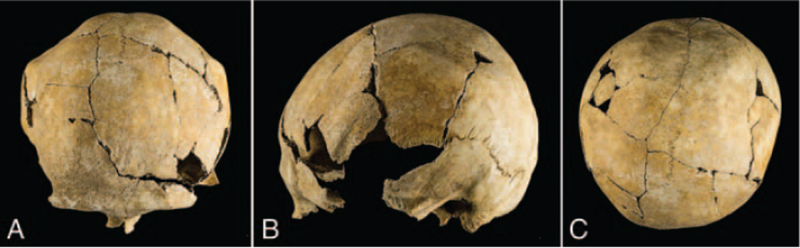
Elliptical and symmetrical depressions located between the temporal line and the sagittal suture of the skull of the individual from Tomb 8: (A) frontal view; (B) left lateral view; (C) superior view.

The parietal bones display 2 elliptical and symmetrical depressions: the right one measured 68.8 × 53.5 mm and the left one measured 73.0 × 57.1 mm. Both lesions show an antero-posterior direction and are located between the temporal line and the sagittal suture (Fig. 4).

**FIGURE 5:**
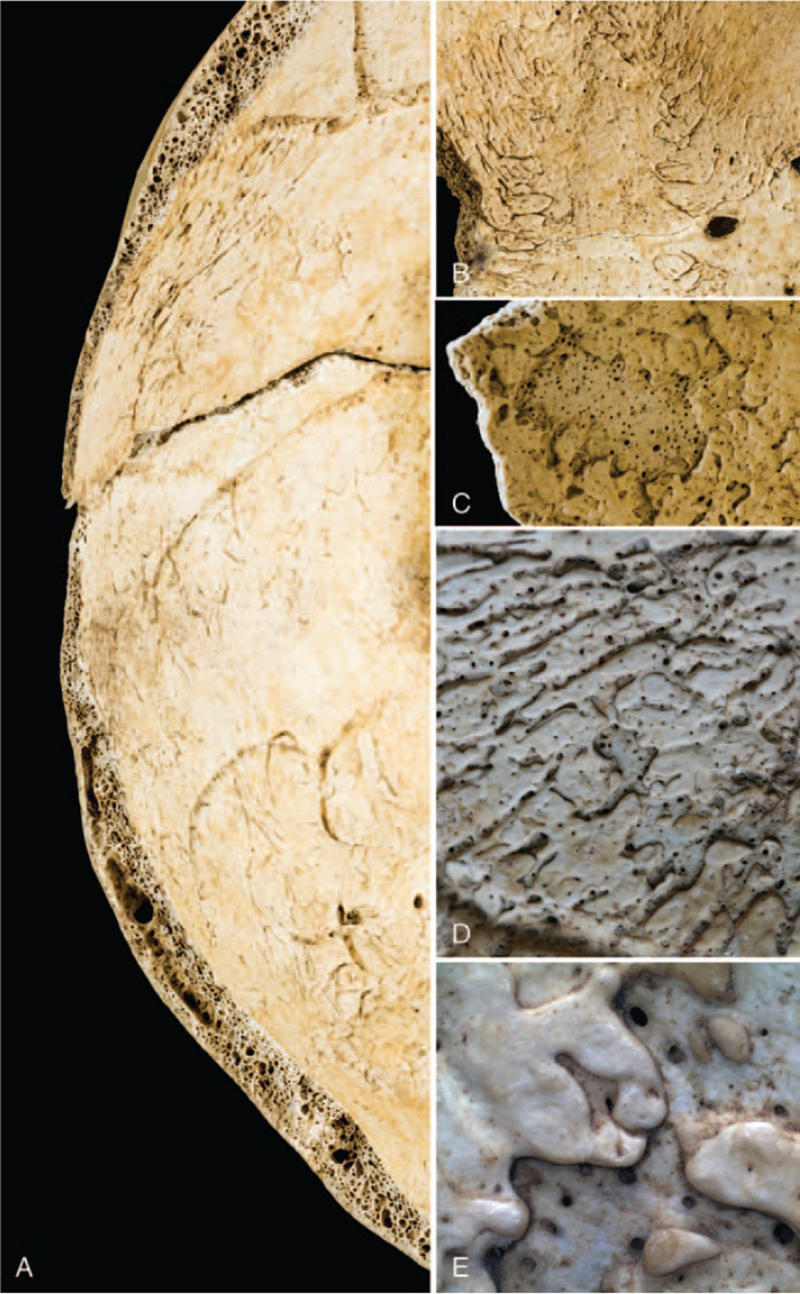
(A) Section of the left parietal with clearly disappearance of diploic tissue and exposure of the inner table; (B) serpentine lesions along the frontal crest and sagittal sinus on the endocranial surface of the frontal bone; (C-D) serpentine lesions and exposure of cribrotic tissue on the endocranial surface of parietal bones; (E) serpentine lesions at 16× magnification.

The right parietal presents a minimum thickness of the cranial theca of 0.75 mm, whereas the left parietal of 0.79 mm. In section, the progressive disappearance of the diploe and the exposure of the internal surface was clearly visible (Fig. 5A). The endocranial surface also presents nonspecific multifocal lesions with a serpentine appearance, located on the frontal and on the parietals, from the frontal crest continuing along the superior sagittal sinus (Fig. 5B-C). The same aspect was also observed at the level of the areas of thinning (Fig. 5D-E).

On the skull, other pathological evidences were recorded. At the maxillary level, the subject is completely edentulous and in the mandible most teeth were lost in life (I2-M3 on the right and M1-M3 on the left side). Indeed, in the alveoli, a clear retraction of the vestibular bone tissue was observed. The mandibular sites of left C and I2 are affected by a cystic granuloma that caused part of the destruction of the cortical bone in the buccal position.

The mandible and palatine bones are also affected by an extremely retracted and thin cortical tissue.

On the post cranial skeleton arthrosis of the spine was highlighted. This condition is particularly expressed on the cervical and lumbar tracts where there was evident osteophytosis of the edges of the vertebral bodies. Besides, an advanced rarefaction of the cancellous bone was visible on the lumbar spine, whereas different lumbar vertebrae present compression fractures.

**FIGURE 6:**
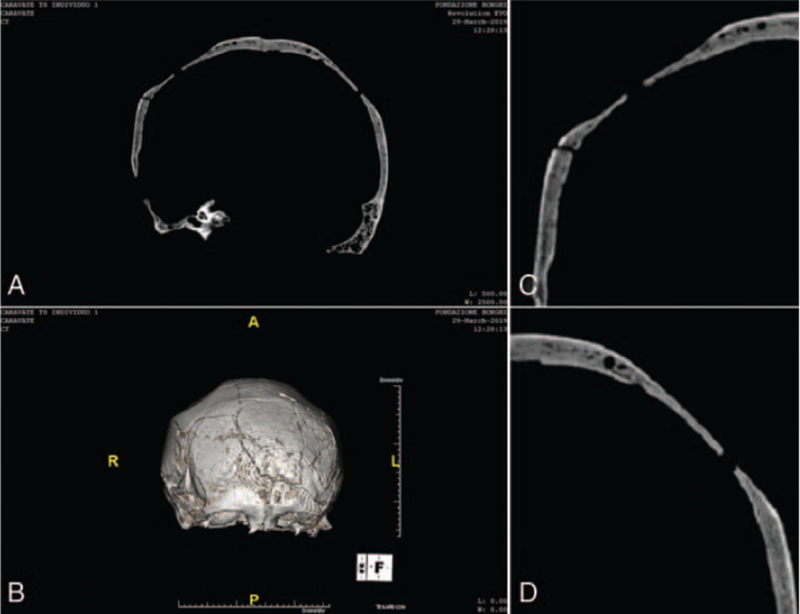
CT coronal vision, symmetrical depression of the parietals, (A) resorption of the external table and of the diploe, (B) 3d reconstruction of the skull, (C) left lateral view, (D) right lateral view. CT, computer tomography.

The computer tomography analyses on the skull allowed us to better understand the lesions and to detect the progressive loss of the diploe and the external bone table, whereas the inner surface appears to be preserved (Fig. 6A-D). Radiological investigations made it possible to detect alterations of the typical curvature of the parietals and strongly expanded venous channels along the sagittal sinus visible in the coronal section.

The histology of an affected portion of parietal bone showed the presence of parallel bone lamellae extended along the entire length of the lower margin of the section.

**FIGURE 7:**
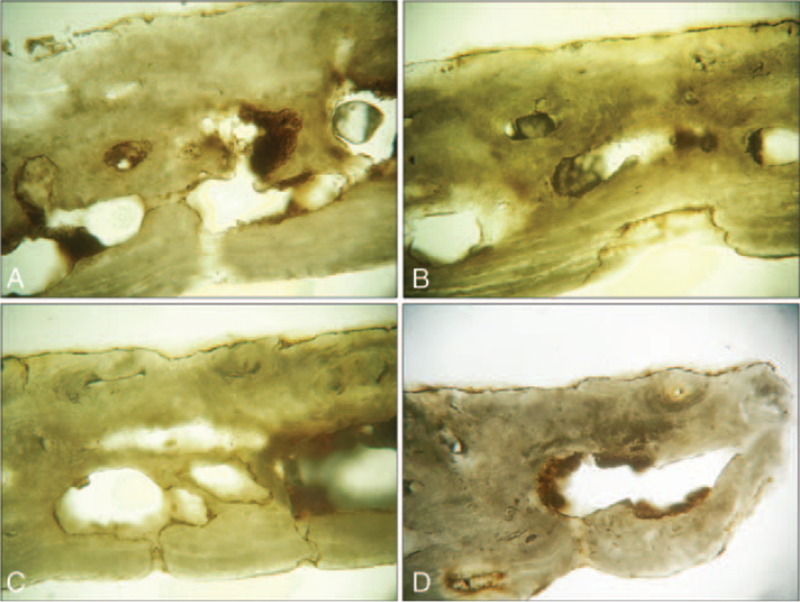
Histological section of an affected portion of parietal bone, observed in normal light (10× magnification). The inner cranial table is on the lower side of the section. The presence of parallel bone lamellae in the lower margin is displayed. In the area above, in the median portion of the section, several osteonic structures immersed in lamellar bone are visible. (A) In the upper margin, no laminated outer layer is observed and several irregularities are noted along the border. In the lower margin of the section, a slight area of remodeling (B) and a thin canal opened in the lamellar bone are displayed (C). An osteonic structure, interested by the erosion and progressive thinning, is observable near the upper margin at the extreme right of the section (D).

The median portion of the slice displays the presence of osteonic systems with visible Haversian canals surround by lamellar bone. In the upper margin of the section, we noted the absence of parallel lamellar bone covering the osteonic lamellae. Several slight irregularities along the margin were instead observed (Fig. 7A-B).

In the lower margin of the section, a slight area of bone loss and remodeling was observed (Fig. 7C-D). Moreover, a thin canal, opened in the lower marginal lamellar bone, was noted (Fig. 7C).

## DISCUSSION

The skeleton shows symmetrical cranial depressions involving both parietal bones in the same location. This case proves to be very interesting, as the cranium appears to be affected by an intense remodeling and loss of substance. As the appearance of this condition respects a symmetrical and bilateral disposition on the parietal bones, our reading of the case refers to a severe condition of biparietal thinning.

Albeit rarely documented, biparietal thinning appears to have been present elsewhere in the past and in today's clinic. Most of the authors agree in affirming that we can observe this condition at all latitudes and with greater frequency in female individuals with a ratio of 1:1.9, although the causes are still debated.^3–6^

Biparietal thinning is a condition that usually affects symmetrically the parietal bones; the lesions generally are oval-shaped and localized between the temporal line and the sagittal suture; the internal surface is usually spared whereas the outer table and diploe are thinned until their disappearance.^1^

Bruyn and Bots described 2 types of biparietal thinning related to each other: flat or grooved.^1^

In 1982, Cederlund classified the degree of the parietal thinning from a radiological point of view. Three stages were observed as follows:

1. thinning is barely recorded and in tomographic images a radiolucent area is highlighted;
2. a considerable thinning is documented, in particular the loss of more than half of the bone substance, even if the diploe is preserved, is noticeable;
3. external table is affected and the total loss of the diploe and of the outer table is recorded.^3^

As some academics highlight, magnetic resonance and computer tomography imaging show the thinning or the absence of the external table, the gradual loss of the diploe and an intact internal surface in the area of the lesion.^6,7,31–33^

For more than 2 centuries researchers have tried to investigate the etiological nature of this condition. The first detailed description of the condition was elaborated by Sandifort in 1783 in *Exercitationes Academicae*.^34^

Different causes have been associated to the condition by several authors. Among others, developmental dysplasia, constant pressure on bones, growth defects, diabetes mellitus, congenital dysplasia of the diploe, primary metastatic tumors, hormonal changes, inflammatory arthropathy associated with trauma, gonadal insufficiency, Gorham disease, senile changes of the temporal artery and simple anatomical variation, osteomyelitis, granulomatous diseases, aseptic necrosis, systemic mastocytosis prolonged steroid therapy, bone aneurysm, and cystic angiomatosis of bone.^11^ Indeed, many researchers linked the biparietal thinning condition to genetic factors^11^, others to vascular causes.^35–37^ Other authors consider the condition age-related,^38,39^ or a consequence of postmenopausal^40,41^ or senile osteoporosis,^9,42^ and atrophy.^3,43^ Moreover, many hypotheses about endocrine^44^ or muscular^45^ origins have been advanced.

Indeed, the incidence of this condition in Egyptian and Moroccan contexts suggests a genetic predisposition,^11,12,46,47^ even if some authors hypothesize the development of the anomaly because of the custom of wearing heavy headgear.^10^ Even the habit of wearing constricting headgear from childhood could cause craniofacial changes.

In fact, it is possible that cranial deformations in childhood lead to incisive bone reactions.^48^

However, only in the most severe cases, they affect the cortex of the skull, usually appearing as porous bone appositions above the ectocranial surface.^48,49^

Furthermore, the loss of cortical tissue during the voluntary modification practice could led to the death of the subject.^48^

Therefore, it is possible to suggest that the evidence recorded by us cannot be link to deformation practices undergone in childhood, especially taking into consideration the skeletal remodeling process that led to the turnover of the skeleton every 10 years.^50^

Moreover, paleopathological literature clearly shows how skull modification patterns differ from those of biparietal dystrophy condition. In fact, the modification of the neurocranium usually took place thanks to constrictions that weighed on the occipital bone, above all, and on the frontal bone, in order to obtain an elongation of the skull in its upper portion.^51^

The use of a mechanical constriction in the parieto superior norm are rare. Finally, the historical and cultural context also allows us to exclude that injuries observed in our sample are the result of voluntary deformation practices.

In fact, although artificial or intentional cranial modification has been a common practice worldwide, ^47,48,49,51,52^ no case regarding the Italian Late Middle Ages has been found.

As we have seen so far, it is evident that the etiology remains to be clarified, but at this point it is important to remember that, among the most acclaimed hypotheses, the senescence seems to be the most convincing.

By the recorded clinical cases, the literature informs us that most cases have a minimum average age of 50, for men, and over 60 for women. It can be suggested that this condition may be related to the reduction or cessation of sex hormone activity.^1^ The hormonal disorder causes osteoporosis, a condition also linked to the lack of osteoclasts, especially in women of advanced age.

It is evident that also for our case, conducting a differential diagnosis is difficult. Although, we can record the presence of bilateral parietal thinning of the third degree, stating to the classification of Cederlund,^3^ and, referring to the classification of Bruyn and Bots,^1^ of the groove type. Moreover, radiological, and histological analysis allowed observing the loss of the outer table and the remodeling and thinning of the diploic layer. Radiological images highlighted the thinning of the diploe until reaching the inner table and the total absence of the outer table. In particular, histological section showed the absence of external lamellar bone, indicating the loss of the outer table in the affected area. Furthermore, osteonic structure seems to be remodeled and interrupted by several irregularities. The widening of the venous furrows in the points of the lesion, clearly visible even on macroscopic analysis, were found without any pathological relevance but rather as the result of the normal senescence process.^42^

It is important to highlight that biparietal thinning is not often considered from a clinical point of view as in mild form usually it has not a pathological significance, except for the potential increased risk of fractures. In fact, cases of epidural hematomas and death caused by trauma, even of lesser entity, are reported in the literature in patients with parietal osteodystrophy.^44–52^

Indeed, our case presents also intracranial lesions, but these may be not related to biparietal thinning, and may rather be attributed to secondary factors. The abnormal vascular impressions on the intracranial surface noted in our case have already been recognized in the past as traces of inflammatory processes or hemorrhages of the meninges.^53,54^

These changes can be caused by a variety of infectious, including tuberculosis, or noninfectious conditions, such as trauma, scurvy, and epidural hematomas.^55–59^

On the other hand, there is often evidence of a correlation between biparietal thinning and senile or postmenopausal osteoporosis.^6,40,41^ A combination of factors in our case seems to partly respond to some hypotheses regarding the etiology of biparietal thinning. The skull, belonging to a mature female, could support the link to biparietal thinning, postmenopausal osteoporosis, and senile osteodystrophy.

However, osteoporosis and senile osteodystrophy as primary causes of this anomaly are less plausible, as these conditions rarely affect cranial bones, rather involving vertebral and femoral bones.^58^

The reduction of the diploe could suggest continuous micro-traumatic stresses of a slight entity but repeated and protracted over time. These micro traumatisms may have caused a chronic inflammatory state with resorption of the epicranial aponeurosis and reduction of trabecular bone.

The micro traumas may have been associated with normal bone degenerative phenomena due to age, probable pregnancies and lactation that may have triggered further inflammatory processes of the meninges or hematoma, which explain the deep grooves of the inner table. They may be caused by the typically female use of carrying loads on the head through circles that weigh mainly on the parietal bones.^60^

In our case, sex and age-at-death of the individual, together with several features observed at the level of post-cranial bones, in particular the pathological fractures of different lumbar vertebrae seem to support the hypothesis that relates biparietal thinning with the ageing process and osteoporosis.^61^

At the same time, the coexistence of lesions on the internal surface of cranial bones, attributable to possible inflammatory processes, leaves open the hypothesis of a micro-traumatic cause at the basis of the parietal thinning.
